# Increased expression of hypoxia-induced factor 1α mRNA and its related genes in myeloid blood cells from critically ill COVID-19 patients

**DOI:** 10.1080/07853890.2020.1858234

**Published:** 2020-12-21

**Authors:** Keiko Taniguchi-Ponciano, Eduardo Vadillo, Héctor Mayani, César Raúl Gonzalez-Bonilla, Javier Torres, Abraham Majluf, Guillermo Flores-Padilla, Niels Wacher-Rodarte, Juan Carlos Galan, Eduardo Ferat-Osorio, Francisco Blanco-Favela, Constantino Lopez-Macias, Aldo Ferreira-Hermosillo, Claudia Ramirez-Renteria, Eduardo Peña-Martínez, Gloria Silva-Román, Sandra Vela-Patiño, Carlos Mata-Lozano, Roberto Carvente-Garcia, Lourdes Basurto-Acevedo, Renata Saucedo, Patricia Piña-Sanchez, Antonieta Chavez-Gonzalez, Daniel Marrero-Rodríguez, Moisés Mercado

**Affiliations:** a Unidad de Investigación Médica en Enfermedades Endocrinas, UMAE Hospital de Especialidades, Centro Medico Nacional Siglo XXI, Instituto Mexicano del Seguro Social, Mexico city, Mexico; b Unidad de Investigación Médica en Enfermedades Oncológicas, UMAE Hospital de Oncología, Centro Medico Nacional Siglo XXI, Instituto Mexicano del Seguro Social, Mexico city, Mexico; c Titular, Coordinación de Investigación en Salud, Instituto Mexicano del Seguro Social, Mexico city, Mexico; d Unidad de Investigación Médica en Enfermedades Infecciosas y Parasitarias, UMAE Hospital de Pediatría, Centro Medico Nacional Siglo XXI, Instituto Mexicano del Seguro Social, Mexico city, Mexico; e Unidad de Investigación Médica en trombosis, hemostasia y aterogénesis, Instituto Mexicano del Seguro Social, Mexico city, Mexico; f Servicio de Medicina Interna, UMAE Hospital de Especialidades, Centro Medico Nacional Siglo XXI, Instituto Mexicano del Seguro Social, Mexico city, Mexico; g Unidad de Investigación Médica en Epidemiología Clínica, UMAE Hospital de Especialidades, Centro Medico Nacional Siglo XXI, Instituto Mexicano del Seguro Social, Mexico city, Mexico; h División de Investigación en Salud, UMAE Hospital de Especialidades, Centro Medico Nacional Siglo XXI, Instituto Mexicano del Seguro Social, Mexico city, Mexico; i Unidad de Investigación Médica en Inmunología, UMAE Hospital de Pediatría, Centro Medico Nacional Siglo XXI, Instituto Mexicano del Seguro Social, Mexico city, Mexico; j Unidad de Investigación Médica en Inmunoquímica, UMAE Hospital de Especialidades, Centro Medico Nacional Siglo XXI, Instituto Mexicano del Seguro Social, Mexico city, Mexico; k Analitek S.A. de C.V., CDMX, México

**Keywords:** COVID-19, SARS-CoV-2, critically ill, scRNAseq, HIF1α, immature myeloid cells

## Abstract

**Background:**

COVID-19 counts 46 million people infected and killed more than 1.2 million. Hypoxaemia is one of the main clinical manifestations, especially in severe cases. HIF1α is a master transcription factor involved in the cellular response to oxygen levels. The immunopathogenesis of this severe form of COVID-19 is poorly understood.

**Methods:**

We performed scRNAseq from leukocytes from five critically ill COVID-19 patients and characterized the expression of hypoxia-inducible factor1α and its transcriptionally regulated genes. Also performed metanalysis from the publicly available RNAseq data from COVID-19 bronchoalveolar cells.

**Results:**

Critically-ill COVID-19 patients show a shift towards an immature myeloid profile in peripheral blood cells, including band neutrophils, immature monocytes, metamyelocytes, monocyte-macrophages, monocytoid precursors, and promyelocytes-myelocytes, together with mature monocytes and segmented neutrophils. May be the result of a physiological response known as emergency myelopoiesis. These cellular subsets and bronchoalveolar cells express HIF1α and their transcriptional targets related to inflammation (CXCL8, CXCR1, CXCR2, and CXCR4); virus sensing, (TLR2 and TLR4); and metabolism (SLC2A3, PFKFB3, PGK1, GAPDH and SOD2).

**Conclusions:**

The up-regulation and participation of HIF1α in events such as inflammation, immunometabolism, and TLR make it a potential molecular marker for COVID-19 severity and, interestingly, could represent a potential target for molecular therapy.

**Key messages**
- Critically ill COVID-19 patients show emergency myelopoiesis.
- HIF1α and its transcriptionally regulated genes are expressed in immature myeloid cells which could serve as molecular targets.
- HIF1α and its transcriptionally regulated genes is also expressed in lung cells from critically ill COVID-19 patients which may partially explain the hypoxia related events.

## Introduction

The coronavirus disease 2019 (COVID-19) epidemic caused by the severe acute respiratory syndrome coronavirus 2 (SARS-CoV-2) has rapidly developed into a devastating pandemic [1]. As of today, the World Health Organization (WHO) has reported more than 46 million persons diagnosed with COVID-19 and over 1.2 million deaths worldwide [2]. It is associated with significant mortality in high risk patients, with poor prognostic features upon admission, such as advanced age, as well as a history of obesity, diabetes and hypertension. The spectrum of the disease is broad, and includes pneumonia, sepsis, and acute respiratory distress syndrome (ARDS) [3]. This complicated course occurs in the context of a cytokine storm characterized by overproduction of TNF, IL6, IL1ß, and G-CSF and generalized vascular hyperpermeability [3]. Viral particles spread through the respiratory mucosa infecting other cells, unleashing a series of immune responses, characterized by a reduction of T and B lymphocytes and an increment of monocytes and neutrophils [4–7]. The pathogenesis of this severe form of COVID-19 is poorly understood.

Hypoxaemia, defined as a decrease in the partial pressure of oxygen is an ominous sign of COVID-19, and it is usually an indicator of disease severity [8,9]. An oxygen saturation above 90% is associated with better outcomes [10]. Over 80% of COVID-19 patients in the intensive care unit have severe hypoxaemia [11]. A kind of “silent hypoxia” in which COVID-19 patients deteriorate rapidly without warning and develop respiratory failure has been described [12]. Hypoxia indicates an imbalance of oxygen delivery to tissues and leads to compromised function, which is quantitatively related to organ, tissue and even cell type. The hypoxia-inducible factors (HIF) are considered master regulators of oxygen homeostasis and are oxygen level sensitive [13,14]. HIF1α is a heterodimeric transcription factor that bind to hypoxia response elements, which participates through the regulation of the expression of several genes in numerous cellular events such as O_2_ sensing, glucose metabolism, lipid metabolism, angiogenesis and other aspects of endothelial biology [14]. Currently, there is scarce information regarding the expression of HIF in patients with severe COVID-19 and its potential involvement in the immunopathogenesis of this condition. Therefore, in the present work we carried out scRNAseq to identify the cell populations present in critically ill COVID-19 patients and to determine the expression of hypoxia-induced factor 1α (HIF1α) and its related genes.

## Materials and methods

### Patients and tissue samples

Blood samples from five critically ill patients with COVID-19 were collected in EDTA-coated tubes. Tissues were collected from patients diagnosed, treated and followed at the Internal Medicine Department of the Hospital de Especialidades, Centro Médico Nacional Siglo XXI of the Instituto Mexicano del Seguro Social in April 2020. A family member of each participating patient signed an informed consent and the study protocol was approved by the Comisión Nacional de Ética e Investigación Científica del Instituto Mexicano del Seguro Social in accordance to the Helsinki declaration. SARS-CoV-2 infection was corroborated by RT-qPCR at an official federal government reference laboratory.

### Sample preparation, scRNAseq library generation and sequencing

Peripheral blood from the five critical COVID-19 patients was collected in EDTA-coated tubes, and immune cells were isolated according to standard centrifugation methods followed by red blood cell lysis.

Chromium Next GEM Single Cell 3′ Reagent Kits v3.1 and the protocol from 10X Genomics was followed as recommended by the manufacturers. Briefly, immune cells were pooled in a single tube and were suspended in 1x phosphate buffered saline (PBS) to 700-1200 cells per µl. Cell suspension was loaded in Chromium Next GEM Chip G and sorted in the Chromium Controller from 10X Genomics. The Cell-Gel Beads in Emulsion (GEMs) were then incubated to generate the barcoded cDNA. cDNA was cleaned using Dynabeads and washed, followed by cDNA amplification and SPRI selection. The retrieved cDNA was enzymatically fragmented, end-repaired, poly-A tailed and ligated. Size selection, adaptor ligation and amplification were done. Sequencing was done using NextSeq 550 System High-Output Kit (300 cycles) in NextSeq 500 system (Illumina) according to 10X Genomics specifications: Read 1 = 28 cycles, Read 2 = 91 cycles, Index 1 = 8 cycles. All quality control steps were carried out using 4200 TapeStation System (Agilent) with High Sensitivity D1000 Screen Tape, whereas the concentration was calculated using Qubit 2.0 Fluorometer with Kit High Sensitivity assays.

### scRNAseq bioinformatic analysis

Partek Flow software was used with scRNAseq toolbox. First the tags were trimmed and then the reads were aligned using STAR 2.7.3a algorithm to human genome hg38. UMI’s were deduplicated and barcode filtered. Following criteria were then applied to each cell, i.e. gene number between 200 and 6000, UMI count above 300 and mitochondrial gene percentage below 20%. To quantify the transcriptome human hg38 Ensembl transcripts release 99 was used. Counts per million, add 1.0 Log 2.0 were the normalization parameters. Healthy donors’ datasets were downloaded from 10X Genomics website and analyzed using Loupe Browser from 10X Genomics. Data has been deposited in Sequence Read Archive hosted by National Centre for Biotechnology Information under accession number PRJNA635580.

### Markers used to circumscribe cell populations

Clusters were categorized by analyzing differentially expressed genes according to previously published data obtained from human samples [15–19].

### Dimensionality reduction and clustering

The filtered and normalized gene-barcode matrix was analyzed by principal components, then graph based and uniform manifold approximation and projection (UMAP) for dimension reduction using default parameters was carried out.

### Cellular alterations analysis

ENRICHR (https://maayanlab.cloud/Enrichr/) was used to understand the biological meaning behind the resulting list of genes, to analyze the gene ontology and pathway information of significantly deregulated genes in the different cellular subsets.

### *In silico* analysis of RNAseq files of cells derived from bronchoalveolar lavage fluid

Paired end fastq files were downloaded from BIGD-GSA and SRI from NCBI. Accession numbers are CRA002390 and SRR10571724, SRR10571730, and SRR10571732. Data files consisted in 3 COVID-19 patients bronchoalveolar lavage fluid (BALF) and 3 bronchoalveolar fluid lavage controls. Fastq files quality control of was performed using FastQC v. 0.11.9. Samples adapter were trimmed with the Cutadapt v tool. 2.10.

BALF samples duplicates were spotted using Picard v. 2.22.4, the RNAseq reads alignments were done using STAR tools v. 2.7.3a, with the parameters previously described [20]. The RNAseq reading count was carried out using the Rsubread package with the annotations of Homo sapiens GRCh.38.99 downloaded from Ensembl. Subsequent analyzes were performed using the stable Gene ID identifier with BioMart annotations: Ensembl Genes 100, Human genes (GRCh38.p13), the identifier was translated to HGNC symbol to facilitate biological interpretation. The BALF counts were subjected to quality control using the NOISeq package, biases associated with length, GC content, RNA composition and batch effect, were identified and removed. BALF samples were processed using full-quantile normalization for GC content and length, finally, the ARSyN function was used to eliminate batch effect. The normalization process was carried out using the NOISeq and EDASeq packages in R. Differentially expressed genes were calculated on the normalized counts using the noiseqbio function of the NOISeq package, using the predefined parameters and filtered by a probability greater than 0.99.

## Results

### Clinical features of critically ill COVID-19 patients

Five male patients with a mean age of 47.8 ± 6.6 years (range 41-57) were studied. Interestingly, four of five patients presented hypoxaemia upon hospital admission to the hospital with O_2_ saturation levels ranging from 40–84% of. The initial SOFA score of the five patients ranged between 2 and 4. All patients had radiological evidence of alveolar occupation and ground glass appearance on imaging studies and required intubation and invasive mechanical ventilation 3 to 7 days after admission as well as hemodynamic collapse that required vasopressor support. All five patients were treated with enoxaparin, hydroxychloroquine, azithromycin or clarithromycin as well as lopinavir and ritonavir; three received high dose glucocorticoids (hydrocortisone or dexamethasone). Three of the five patients died of ARDS and multiorgan failure (Table 1).

**Table 1.** Characteristics of critically ill patients with COVID-19.

	Patient 1	Patient 2	Patient 3	Patient 4	Patient 5
Age, yrs	57	46	43	41	52
Gender	M	M	M	M	M
% Oxygen saturation at admission	40	50	NA	57	84
SOFA score at admission	4	2	2	2	2
BMI, kg/cm^2^	29	26.3	31	24	29
Comorbidities	OW	None	DM, OW	None	DM, OW
Time from admission to intubation, days	4	3	5	3	5
Leukocytes per mm^3^	9130	8760	7760	8900	5060
Lymphocytes, x 10^3^/mm^3^	650	520	160	**1130**	**1600**
Hb, g/dL	16.4	15.9	17	12.9	15.8
Platelets, x 10^3^/mm^3^	193	357	380	523	169
CRP, mg/L	23.6	35	11.9	27	11.6
Procalcitonin, ng/mL	2.32	1.87	0.07	0.22	0.33
D-Dimer, ng/mL	2410	3320	1100	2980	2020
Fibrinogen, mg/dL	834	788	753	789	773
Final outcome	Dead	Dead	Dead	**Alive**	**Alive**

OW: overweight; DM: type 2 diabetes mellitus; BMI: body mass index; SOFA: sequential organ failure assessment; NA: not available; M: male.

### Immature myeloid cell populations in critically ill COVID-19 patients

**Figure 1.**
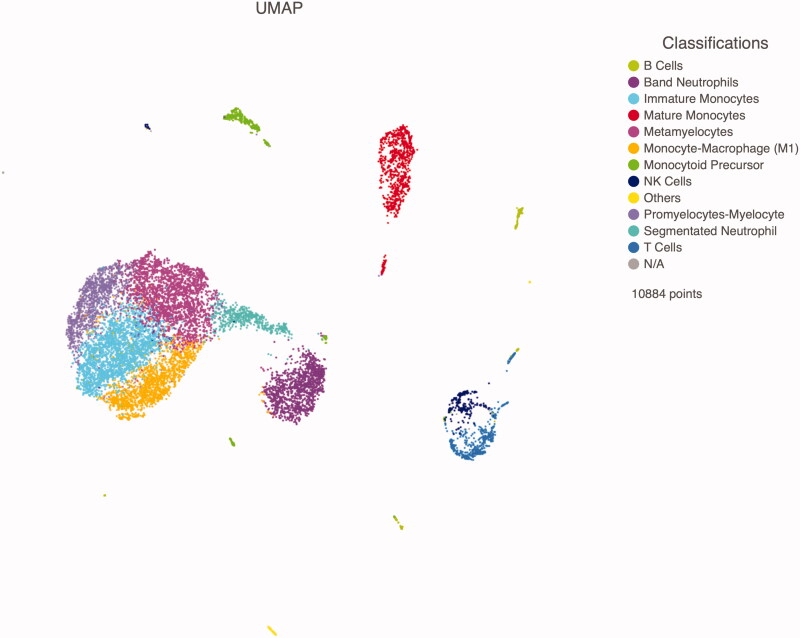
Uniform manifold approximation and projection (UMAP) showing the identification of 12 cell clusters in critically ill COVID-19 patients. Immature myeloid populations, such as band neutrophils, metamyelocytes, promyelocytes-myelocytes, monocytoid precursor, and immature monocytes, as well as mature lineages, such as segmented neutrophils, mature monocytes and monocyte-macrophages are observed.

We have previously reported that, as compared to healthy adults, lymphoid cell subsets, such as B and T lymphocytes as well as NK cells, are present in low quantities in critically ill COVID-19 patients, whereas cells of myeloid origin predominate. Interestingly, immature myeloid cell populations, such as band neutrophils, metamyelocytes, promyelocytes-myelocytes, monocytoid precursor, and immature monocytes prevailed. Mature lineages such as segmented neutrophils, mature monocytes and monocyte-macrophages were also observed (Figure 1) [20].

### Altered cellular pathways in immune cells from critically ill COVID-19 patients

Myeloid cell population subsets presented alterations in: (1) neutrophil-mediated immunity, (2) cellular response to type I interferon and (3) cell cycle control, DNA and RNA processing. The lymphoid cellular subs*et al*terations were mostly related to ribosome biogenesis as well as RNA processing and modification. Along with the gene ontology analysis the algorithm compares the gene list input data with publicly available information and thus, our gene set lists were in accordance with COVID-19 data (Supplementary Figure 1).

### HIF1α expression in leukocytes from critically ill COVID-19 patients

**Figure 2.**
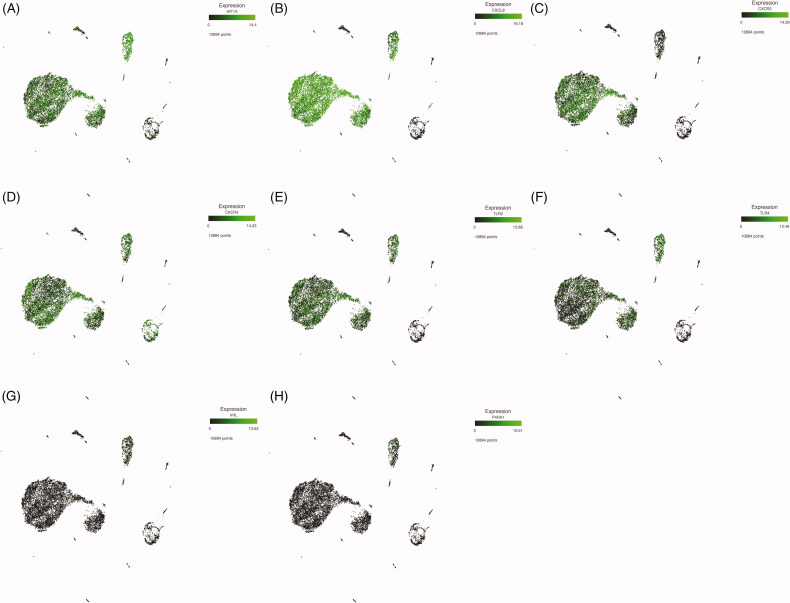
Expression levels of HIF1α and transcriptionally regulated target genes in peripheral blood cell lineages. HIF1α (A), CXCL8 (B), CXCR2 (C), CXCR4 (D), TLR2 (E), TLR4 (F) VHL (G) and P4HA1 (H) mRNA’s expression.

Once the blood cell populations were identified, we looked for HIF1α gene expression. As shown in Figure 2(A), HIF1α gene was expressed in all myeloid lineages to a greater extent than in lymphoid cells. This was particularly evident in the mature monocyte population. Since HIF1α is a transcription factor that acts as a *trans* regulator, we searched for HIF1α-regulated genes potentially involved in COVID-19 immunity. Among these immune related genes, we found an increased expression of CXCL8 or Interleukin-8, a chemokine involved in the migration of mature neutrophils to the site of infection in most myeloid cell subsets, and almost no expression in lymphoid populations and monocytoid precursors (Figure 2(B)). In keeping with the increased expression of CXCL8, the genes for chemokine receptors CXCR2 (Figure 2(C)), CXCR4 (Figure 2(D)), as well as CXCR1, were also expressed at increased levels in most myeloid lineages. It is noteworthy that lymphoid cells did express the CXCR4 gene, but showed no expression of the CXCR2 gene, which can explain the exacerbated inflammatory response characteristic of these patients [21]. Interestingly, we found expression of Toll like receptor-2 and −4 (TLR2 and TLR4) in most myeloid populations, which could be related to SARS-CoV-2 sensing (Figure 2(E,F)).

**Figure 3.**
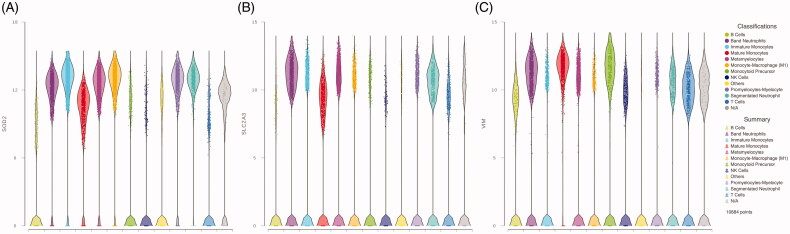
Violin plots from HIF1α transcriptionally regulated genes. (A–C) The expression of SOD2, SLC2A3 and VIM, respectively, in peripheral blood cells from critically ill COVID-19 patients.

Along with the identified genes regulated by HIF1α, we found expression of genes related to metabolism such as solute carrier family 2 member 3 (SLC2A3) also known as GLUT3, 6-phosphofructo-2-kinase (PFKFB3), phosphoglycerate kinase 1 (PGK1) and glyceraldehyde-3-P-dehydrogenase (GAPDH) (Figure 3). GAPDH is related to neutrophil survival through the inhibition of programed cell death [22].

We also found expression of superoxide dismutase 2 (SOD2), involved in the metabolism of reactive oxygen species; vimentin (VIM), a type III intermediate filament and plasminogen activator urokinase receptor (PLAUR), which is related to plasminogen activation.

Considering that HIF1α function is controlled by different factors, we also evaluated the expression of the Von Hippel Lindau (VHL) and prolyl-4-hydroxylase (P4HA1) genes, two of the main inhibitors of HIF1α function. Indeed, these two molecules are involved in the ubiquitination and degradation of HIF1α. In keeping with the increased expression of HIF1α and target genes observed, we found that neither VHL nor P4HA1 were expressed by the peripheral blood cells of critically ill COVID-19 patients (Figure 2(G,H)).

**Figure 4.**
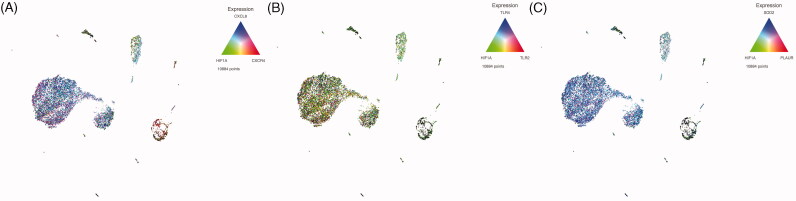
Simultaneous expression of HIF1α and target genes on the same single cell. (A) The expression of HIF1α (green), CXCL8 (blue) and CXCR4 (red). (B) The expression of HIF1α (green), TLR2 (red) and TLR4 (blue), and panel C) the expression of HIF1α (green), SOD2 (blue) and PLAUR (red).

We next evaluated the potential interaction between HIF1α and their transcriptional targets by assessing the simultaneous presence of their mRNAs in the same single cells (Figure 4). As shown in Figure 4(A), a significant proportion of myeloid cells co-expressed HIF1α and CXCL8. We also observed a predominance of cells co-expressing HIF1α and TLR2 (Figure 4(B)) as well as HIF1α and SOD2 (Figure 4(C)). It is noteworthy that among the different myeloid subsets, mature monocytes were the ones that exhibited co-expression of most of the analyzed genes.

**Figure 5.**
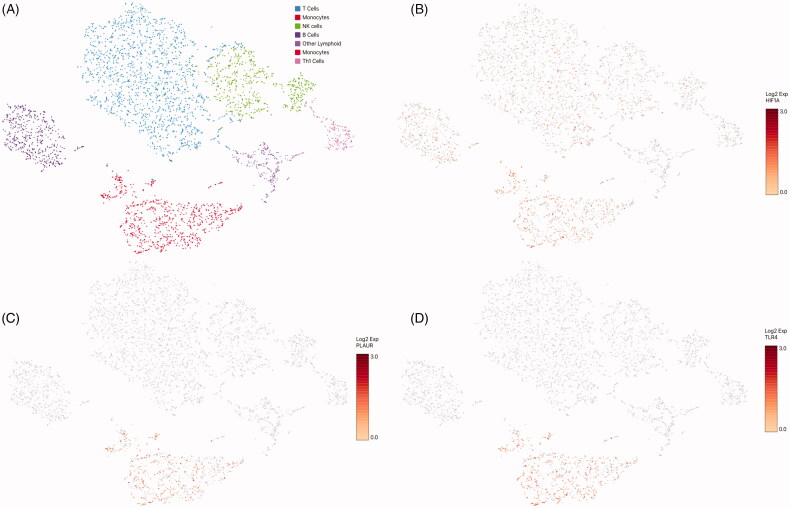
Cell populations and HIF1α gene expression identified in peripheral blood cells from healthy individuals. (A) The cell subsets identified in healthy donors. (B–D) The expression of HIF1α, PLAUR and TLR4 mRNA, respectively.

Finally, we evaluated the expression of HIF1α and its transcriptional targets in peripheral blood cells from healthy donors. To do so, we analyzed 10X Genomics publicly available datasets. We observed that HIF1α expression was lower as compared to the expression observed in COVID-19 patients (Figure 5). Similar results were observed for genes such as CXCL8, CXCR2, PLAUR, TLR4 and SOD2 (Figure 5).

### HIF1α expression and its transcriptionally regulated genes in SARS-CoV-2 infected lungs

**Figure 6.**
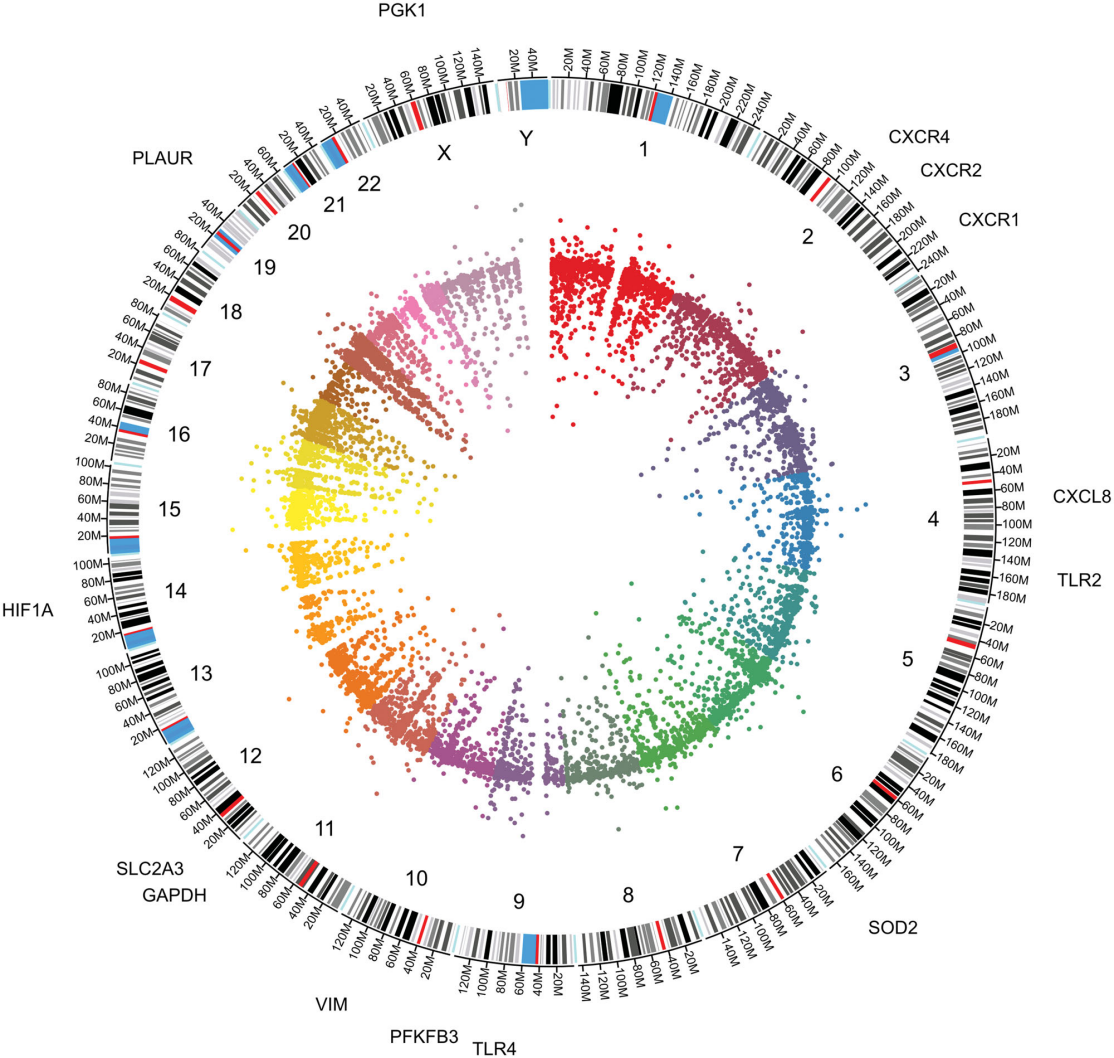
Circos plot from the BALF cells transcriptome from COVID-19 patients. Inner ring is the scatter plot representing each transcript identified followed by chromosome number and size, the outer ring present HIF1α-related gene expression.

Lung is one of the main organs affected by SARS-CoV-2 infection. We have previously described the alteration of hyaluronan, glycosaminoglycan and mucopolysaccharides metabolism which could, in part, explain the presence of a viscous fluid found in the interstitial space that reduces lung compliance and thus, effective ventilation [23]. Lung cells from COVID-19 patients show expression of HIF1α, TLR2, TLR4, PFKFB3, CXCR1, −2 and −4, SOD2, PLAUR (Figure 6) and other genes expressed in immature myeloid cells.

## Discussion

Using a scRNAseq approach we have found a distinctive predominance of immature cell lineages of myeloid origin and a reduced number of lymphoid lineages in patients with life-threatening respiratory distress syndrome due to SARS-CoV-2 infection, which is in accordance with recently reported data [6–8]. Collectively, our results are in accordance with similar observations were severe COVID-19 patients presented marked changes in immune cell composition and phenotype, reflecting an emergency granulopoiesis [24]. It is noteworthy that a similar blood profile has been observed in patients with acute parvovirus B19, cytomegalovirus and Epstein-Barr viral infection [20]. In line with this, it has been reported in severe COVID-19 patients, as well as, some bacterial infections that a high neutrophil-to-lymphocyte ratio is associated to multi-organ damage and mortality [25].

Hypoxia and inflammation are unequivocally linked [26] and are two of the main physiological consequences of SARS-CoV-2 infection, particularly in severe cases. In this study we present scRNAseq data regarding HIF1α-related gene expression in peripheral blood leukocytes from critically ill COVID-19 and characterized the different cell subpopulations. HIF1α is a heterodimeric transcription factor sensitive to oxygen and induced under hypoxic conditions [27]. The HIF1α *trans* element can regulate the expression of CXCL8 [28], CXCR1, CXCR2 [29] and CXCR4 [30]. CXCL8 expression can be stimulated by interleukin (IL) 6, TNFα, hypoxia [31] and viral infection [32] in cells such as monocytes, neutrophils, epithelial cells and fibroblasts [33]. CXCL8 is a chemokine that exerts its pro-inflammatory functions throughout the CXCR1 and CXCR2 receptors. CXCL8 and its receptors contribute to pathogen elimination, through the transient activation of ERK, AKT, SRC and FAK leading to activation of neutrophils [32]. The expression of CXCL8, which is present in COVID-19 patients, is considered a potential prognostic factor in acute respiratory distress syndrome (ARDS) [34] and lung injury [35].

The SARS-CoV-2 viral entry depends upon binding of viral spike (S) protein to the host cell surface protein angiotensin-converting enzyme 2 (ACE2) [36]. Under physiological conditions, there is an equilibrium between ACE1 and ACE2. However, under hypoxic conditions the ACE2 gene is negatively regulated by HIF1α. It has been suggested that increased levels of ACE2 positively correlate with COVID-19, thus the stabilization of HIF1α which in turn, down regulates the ACE2 gene, could improve the outcomes in COVID-19 patients [37,38].

The immunopathological outcomes are most likely induced by the host-virus interaction. The interaction between viral antigen and host immune cells results in an exacerbated inflammatory response [39]. In the present study, we also found high expression of both TLR2 and TLR4 genes in peripheral blood leukocytes of severe COVID-19 cases. The viral Spike protein can be recognized by TLR2 [40] and TLR4 [39]. Upregulation of TLR2 and TLR4 has been found in the context of infections with the original SARS-CoV [41]. TLR4 constitutes one of the most efficient innate immune receptors, triggering pro-inflammatory responses after binding to the pathogenic ligand, and this interaction could be useful for developing drugs [39].

Metabolic reprograming of innate immune cells occurs during hyperinflammatory states. Immune cells contribute to systemic changes in metabolism by altering their metabolic profiles in response to the immunological state. Therefore, therapeutic modulation of immune cell metabolism could alter the inflammatory state and thus improve patient prognosis [42]. Inflammation and hypoxia are inherently linked, and hypoxia is a well-known glycolysis driver as oxygen deficit results in limited OXPHOS [43]. Previous studies have shown that the molecular mechanisms underlying the switch from OXPHOS to glycolysis during innate immune cell response require HIF1α [42]. Among the HIF1α responsive genes, we found expression of those related to carbohydrate metabolism, such as SLC2A3/GLUT3, PFKFB3, PGK1 and GAPDH [44]. HIF signalling pathway activation in neutrophils results in an increased survival of these cells, β2 integrin expression, production of antimicrobial peptides and glycolysis. Neutrophils use high rates of Warburg-like glycolysis for ATP generation. The absence of HIF1α leads to reduced ATP pools resulting in a profound impairment of the inflammatory response [45]. HIF1α can also regulate nitric oxide production, pentose phosphate pathway, OXPHOS and arginase metabolism [42]. Overall, immunometabolism is now considered an indispensable regulator of immunity, with HIF1α playing a central role, modulating the function of various immune cell subsets [45]. The expression of HIF1α has been previously found to be a sepsis marker [42].

HIF1α participates in the regulation of a plethora of cellular events such as metabolism of ROS trough the regulation of SOD2 [46], the regulation of cytoskeleton trough VIM type III filament, which also participates in inflammation [47], and PLAUR which activates plasminogen and activates a cascade of extracellular proteases [48]. Interestingly, the expression of this gene could be used as a predictor of severe respiratory failure [49] which is consistent with our results.

## Conclusion

In conclusion, in the present study, we have demonstrated the expression of HIF1α and its transcriptionally regulated genes, in myeloid cells, including both mature and immature subsets, present in peripheral blood of critically ill COVID-19 patients. The up-regulation and participation of HIF1α in relevant events such as inflammation, immunometabolism, and TLR supports its use as molecular marker for COVID-19 severity and as a potential candidate for targeted therapy.

## Data Availability

Data have been deposited in Sequence Read Archive hosted by National Centre for Biotechnology Information under accession number PRJNA635580.
